# Histopathological differences in pediatric duodenogastric reflux: a comparative study

**DOI:** 10.1007/s00431-025-06163-z

**Published:** 2025-05-14

**Authors:** Sevde Nur Türker, Zeren Barış, Nazlı Sena Şeker, Yusuf Aydemir

**Affiliations:** 1 Faculty of Medicine, Department of Pediatrics, Eskişehir Osmangazi University, Eskişehir, Turkey; 2 Faculty of Medicine, Department of Pediatric Gastroenterology, Eskişehir Osmangazi University, Eskişehir, Turkey; 3 Faculty of Medicine, Department of Pathology, Eskişehir Osmangazi University, Eskişehir, Turkey

**Keywords:** Duodenogastric reflux, Pediatric, Histopathology, Foveolar hyperplasia, Fibrosis, Bile reflux

## Abstract

The histopathological effects of duodenogastric reflux (DGR) in children remain poorly described. This study aimed to evaluate and compare the gastric histopathological findings of pediatric patients with endoscopically confirmed DGR gastritis and those without, to identify potential morphological changes associated with bile reflux in childhood. This retrospective study compared children with endoscopically confirmed DGR to age- and sex-matched controls without DGR. Gastric biopsy samples were re-evaluated by a single pathologist blinded to clinical data. Histopathological features, including inflammation severity, activity, fibrosis, vascular congestion, edema, foveolar hyperplasia, the presence of *Helicobacter pylori*, lymphoid aggregates, reactive gastropathy, intestinal metaplasia, and glandular atrophy were compared. Logistic regression was used to identify significant predictors of DGR. A total of 73 patients with DGR and 65 controls were included. Fibrosis (60.2% vs. 9.2%, *p* < 0.001), congestion (63.0% vs. 27.7%, *p* < 0.001), foveolar hyperplasia (32.9% vs. 6.2%, *p* < 0.001), and edema (24.7% vs. 6.2%, *p* = 0.003) were significantly more common in the DGR group. Logistic regression identified foveolar hyperplasia (OR 10.67), edema (OR 9.01), fibrosis (OR 6.98), and congestion (OR 5.85) as independent predictors of DGR. *Conclusion*: Fibrosis, congestion, foveolar hyperplasia, and edema are significantly associated with DGR in pediatric patients and may serve as supportive histological markers for diagnosis.

**Table:** 

**What is Known:** • *DGR in children lacks a standardized diagnostic method, with endoscopy and histopathology being commonly used.* • *Histopathological features such as foveolar hyperplasia and fibrosis are known in adults but less studied in children.*	
**What is New:** • *This study identifies fibrosis, congestion, foveolar hyperplasia, and edema as significant histopathological markers in pediatric DGR.* • *It suggests that endoscopic findings, combined with histopathology, can aid in the diagnosis of DGR in children*.

**What is Known:**

• *DGR in children lacks a standardized diagnostic method, with endoscopy and histopathology being commonly used.*

• *Histopathological features such as foveolar hyperplasia and fibrosis are known in adults but less studied in children.*

**What is New:**

• *This study identifies fibrosis, congestion, foveolar hyperplasia, and edema as significant histopathological markers in pediatric DGR.*

• *It suggests that endoscopic findings, combined with histopathology, can aid in the diagnosis of DGR in children*.

## Introduction

Duodenogastric reflux (DGR) refers to the backflow of duodenal contents, such as bile and pancreatic secretions, into the stomach. While transient bile reflux may occur physiologically, persistent exposure of the gastric mucosa to these alkaline substances can result in mucosal injury and inflammation, collectively referred to as DGR disease [1].

In recent years, a noticeable increase in pediatric DGR has been observed, potentially influenced by changing dietary habits, increased consumption of processed foods, and irregular meal patterns [1, 2]. Advances in endoscopic techniques and greater clinical awareness may also contribute to the growing number of diagnoses. Although DGR is well-documented in adults—especially following gastric surgery—its recognition and clinical implications in children remain limited [3, 4]. In the pediatric population, DGR may develop without structural abnormalities, and nonspecific symptoms and the lack of standardized criteria often complicate diagnosis [1].

Histopathological examination of gastric biopsies may offer valuable insights into the mucosal effects of bile reflux. Previous studies in animal models and adult patients have associated DGR with characteristic features such as foveolar hyperplasia, edema, vascular congestion, and fibrosis [5, 6]. However, pediatric data are scarce, and the diagnostic significance of these findings in children is unclear [7].

This study aims to identify histopathological features that are more frequently observed in pediatric patients with DGR, which may provide supporting evidence in evaluating suspected cases.

## Methods

### Study design and patient selection

This retrospective study was conducted at Eskişehir Osmangazi University Faculty of Medicine, Department of Pediatric Gastroenterology, between 2020 and 2023. Pediatric patients under 18 years of age who underwent upper gastrointestinal endoscopy due to dyspeptic complaints were screened.

**Fig. 1:**
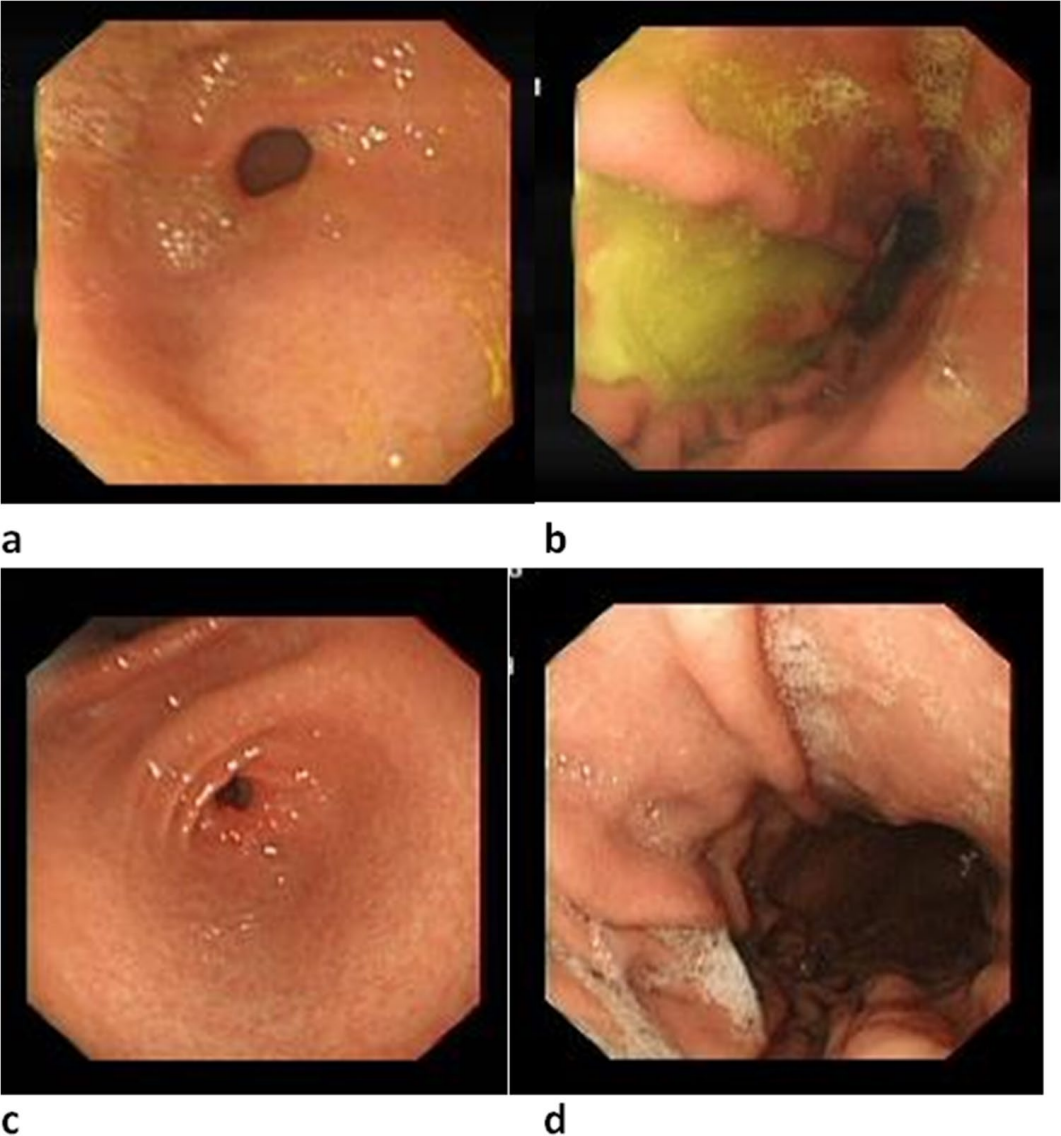
Endoscopic findings in pediatric patients. **a** Antrum and **b** corpus endoscopic images in a patient with duodenogastric reflux: the mucosa is hyperemic, edematous, and covered with thick bile. **c** Antrum and **d** corpus endoscopic images in a patient from the control group: the mucosa is hyperemic and edematous, without bile reflux

The endoscopies of the patients were performed using an EVUS X1 CV-1500 or EVIS CV-260 SL Endoscopy device (Olympus, Japan) in the Endoscopy Unit. Oral and written consent was obtained from the families prior to the endoscopy. All patients fasted for 8 h before the endoscopy, and endoscopic procedures were performed after sedation with midazolam (0.1 mg/kg) and propofol (1 mg/kg). During the endoscopy, the esophagus, cardia, fundus, corpus and antrum regions of the stomach, and the duodenum were examined in detail by two pediatric gastroenterologist. The biopsy samples were taken routinely from the esophagus, corpus, antrum, and duodenum (two samples from each region, with a third biopsy from the antrum taken to evaluate the presence of *Helicobacter pylori* by rapid urease test). During the endoscopic examination, gastritis findings such as hyperemia, fragility, edema, gastric and duodenal ulcers and erosions, masses, hemorrhage, hiatal hernia, strictures and stenosis in the inferior esophageal sphincter, bile pool, duodenogastric bile reflux, and shape and function of the pyloric sphincter were noted. The endoscopic data of the patients and endoscopic images of each examined region were recorded. A pediatric gastroenterologist retrospectively reevaluated endoscopic reports and images of the patients. Patients with endoscopic findings of gastritis and duodenogastric bile reflux with thick bile or bile stain were included in DGR disease group (Fig. 1a and 1b). Patients who were reported to have bile in stomach but without endoscopic findings of gastritis were eliminated from the DGR disease group in the reevaluation process. Those who underwent prior *H. pylori* eradication treatments, prior gastric or bile duct surgery, prior endoscopic retrograde cholangiopancreatography procedures, those who received corticosteroids, nonsteroidal antiinflammatory drugs, antibiotics or proton pump inhibitors within 1 month before endoscopy, and those who had systemic comorbid diseases (e.g.,diabetes, hypertension) or coexisting gastrointestinal diseases (e.g., peptic ulcer, eosinophilic gastritis, inflammatory bowel disease) were excluded from the study.

The control group consisted of age- and sex-matched pediatric patients who underwent endoscopy for dyspeptic complaints, who has endoscopic findings of gastritis but did not show any evidence of bile reflux during the procedure (Fig. 1c and 1d). Same exclusion criteria were applied for the control group.

Between 2020 and 2023, a total of 298 pediatric patients underwent upper gastrointestinal endoscopy due to dyspeptic symptoms. Of these, 122 patients were found to have visible bile reflux in the stomach. After applying inclusion and exclusion criteria, 73 patients with sufficient data and endoscopically confirmed bile reflux disease were included in the DGR disease group, 65 patients were included in the control group.

The study was conducted following institutional ethical guidelines and received approval from the local Research Ethics Committee in accordance with the Declaration of Helsinki (approval date: 11/04/2023, No. 20).

Gastric biopsy samples that were obtained from the antrum during endoscopy were retrospectively re-evaluated by a single experienced gastrointestinal pathologist blinded to clinical and endoscopic findings. Routine hematoxylin and eosin (H&E) staining was performed for all samples. Periodic acid–Schiff and Alcian blue (PAS–AB) staining was used to assess intestinal metaplasia, and immunohistochemistry was employed for detection of *H. pylori*. The histopathological parameters analyzed according to the Updated Sydney System, included the following: inflammation severity (mild, moderate, severe), severity of activity (absent, mild, moderate, severe), presence of fibrosis, degree of fibrosis (mild, moderate, severe), vascular congestion, edema, foveolar hyperplasia, presence of *H. pylori*, lymphoid aggregates, reactive gastropathy, intestinal metaplasia, and glandular atrophy [8, 9]. Each parameter was compared between the DGR disease and control groups.

Statistical analyses were performed using IBM SPSS Statistics version 22.0 (IBM Corp., Armonk, NY, USA). Categorical variables were presented as frequencies and percentages, while continuous variables were expressed as mean ± standard deviation. Chi-square or Fisher’s exact test was used for comparison of categorical variables. Student’s *t* test or Mann–Whitney *U* test was used for continuous variables, depending on data distribution. Variables found to be significant in univariate analyses were entered into a logistic regression model to identify independent predictors of DGR disease. A *p* value < 0.05 was considered statistically significant.

## Results

### Demographic characteristics

A total of 138 pediatric patients were included in the study: 73 in the DGR disease group and 65 in the control group. The mean age of the DGR group was 14.5 ± 2.4 years, and 71.2% were female. The control group was matched for age and sex (mean age 14.5 ± 2.0 years, and 56.9% were female), and no statistically significant differences were observed in demographic characteristics between the groups (Table 1).

**Table 1:** Demographic findings in duodenogastric reflux and control groups

Demographic finding	Control	DGR	*p* value
Age (mean ± SD/median)	14.5 ± 2	14.5 ± 2.4	> 0.05
Gender			> 0.05
Female	37 (56.9%)	52 (71.2%)	
Male	28 (43.1%)	21 (28.8%)	

### Histopathological findings

**Fig. 2:**
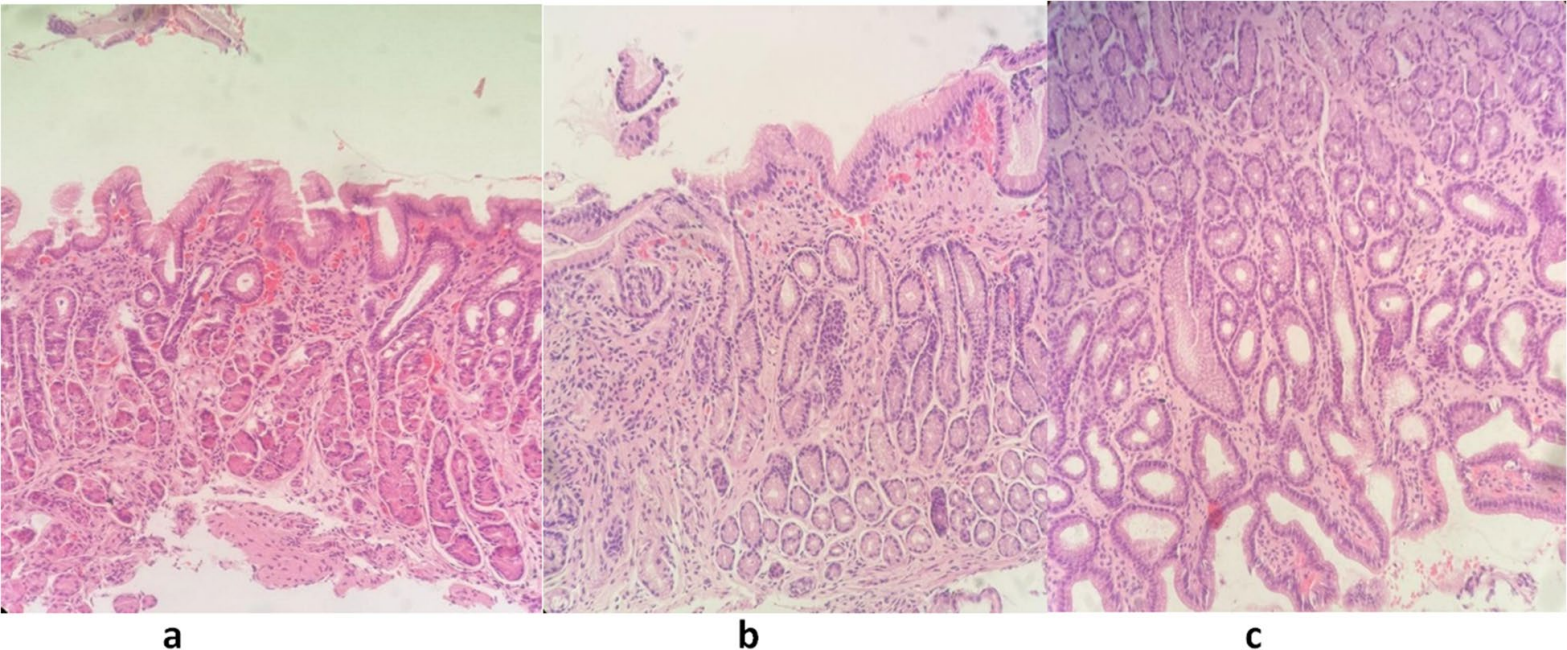
Histopathological findings in duodenogastric reflux. **a** Prominent vascular structures with congestion in the lamina propria, foveolar hyperplasia on the surface, and mild fibrosis. **b** Moderate fibrosis, congested vascular structures, and a mild area of inflammation. **c** Foveolar hyperplasia and mild chronic inflammation

Histopathological features were compared between the DGR and control groups (Table 2). Fibrosis was significantly more frequent in the DGR group (60.2%) compared to the control group (9.2%) (*p* < 0.001). Vascular congestion (63.0% vs. 27.7%, *p* < 0.001), foveolar hyperplasia (32.9% vs. 6.2%, *p* < 0.001), and edema (24.7% vs. 6.2%, *p* = 0.003) were also significantly more common in the DGR group (Fig. 2). Other findings, such as inflammation severity, lymphoid aggregates, reactive gastropathy, metaplasia, and atrophy, showed no significant differences between groups.

**Table 2:** Histopathological features in duodenogastric reflux and control groups

Pathological findings	Control, *n* (%)	DGR, *n* (%)	*p* value
Inflammation severity			0.096
Mild	57 (87.7)	56 (76.7)	
Moderate	8 (12.3)	13 (17.8)	
Severe	0	4 (5.5)	
Fibrosis			** ≤ 0.0001**
Absent	59 (90.8)	44 (60.2)	
Mild	6 (9.2)	28 (38.4)	
Moderate	0	1 (1.4)	
Severe	0	0	
Severity of activity			0.1
Absent	65 (100)	70 (94.5)	
Mild	0	1 (1.4)	
Moderate	0	2 (4.1)	
Severe	0	0	
Congestion	18 (27.7)	46 (63)	** ≤ 0.0001**
Foveolar hyperplasia	4 (6.2)	24 (32.9)	** ≤ 0.0001**
Edema	4 (6.2)	18 (24.7)	**0.003**
Presence of *H. pylori*	2 (3.1)	8 (11)	0.076
Presence of lymphoid aggregates	1 (1.5)	3 (4.1)	0.371
Reactive gastropathy findings	0	3 (4.1)	0.1
Metaplasia	0	0	─
Atrophy	0	0	─

Although the presence of *H. pylori* was higher in the DGR group (11.0%) compared to the control group (3.1%), the difference was not statistically significant (*p* = 0.076). The inflamation was more severe, severity of activity was higher, and lymphoid aggregates were more common in patients with *H. pylori* compared to patients without *H. pylori* in total DGR and control groups (*p* < 0.0001, *p* < 0.0001, and *p* = 0.001, respectively). Foveolar hyperplasia, vascular congestion, edema, and fibrosis showed no significant difference between patients with and without *H. pylori.*

### Logistic regression analysis

Multivariate logistic regression was performed to identify independent histopathological predictors of DGR (Table 3). Foveolar hyperplasia was found to be the strongest predictor (OR 10.67, 95% CI 2.76–41.15, *p* = 0.001), followed by edema (OR 9.01, 95% CI 2.29–35.44, *p* = 0.002), fibrosis (OR 6.98, 95% CI 2.23–21.84, *p* < 0.001), and congestion (OR 5.85, 95% CI 2.24–15.23, *p* < 0.001).

**Table 3:** Logistic regression analysis of histopathologic predictors for duodenogastric reflux

Histopathologic predictor	Odds ratio (95% CI)	*p* value
Foveolar hyperplasia	10.67 (2.76–41.15)	0.001
Edema	9.01 (2.29–35.44)	0.002
Congestion	5.85 (2.24–15.23)	0.000
Fibrosis	6.98 (2.23–21.84)	0.000

## Discussion

This study investigated histopathological differences between pediatric patients with endoscopically confirmed gastritis, one group associated with DGR and the other without. Our findings demonstrate that foveolar hyperplasia, vascular congestion, lamina propria edema, and fibrosis are significantly more common in children with DGR, and these features emerged as independent predictors in multivariate analysis. These results are consistent with the histopathological profile of bile-induced gastric injury previously described in adult literature and, to a lesser extent, in pediatric studies [1, 3, 6, 7].

In pediatric populations, the histological consequences of DGR have historically been under-recognized. Early studies, such as those by Szarszewski et al., suggested that DGR was common but not necessarily associated with significant mucosal injury in children, and they reported no clear increase in inflammation or *H. pylori* density in DGR patients compared to controls [10]. Similarly, Hermans et al. described minimal histological changes in children with documented bile reflux despite significant reflux demonstrated on Bilitec monitoring [11]. These observations led to a long-standing belief that pediatric gastric mucosa might be more resistant to bile injury or that DGR in children was predominantly a functional condition without histologic correlates.

However, more recent pediatric studies have challenged this assumption. Zhang et al. systematically evaluated gastric biopsies in *H. pylori*-negative children with confirmed bile reflux and reported that foveolar hyperplasia, edema, and vascular congestion were prominent histopathological features [7]. Our results reinforce these findings, particularly by confirming the diagnostic value of these histological changes in a larger pediatric cohort.

Among the observed features, foveolar hyperplasia is widely accepted as a hallmark of chemical injury in the gastric mucosa. It represents an adaptive response to irritants such as bile or NSAIDs, characterized by elongation and tortuosity of the gastric pits, epithelial hyperplasia, and nuclear atypia without significant mitotic activity [3]. In our study, foveolar hyperplasia was found in nearly one-third of DGR patients, a rate significantly higher than in the control group. This is in agreement with pediatric and adult studies, where the frequency of this finding correlates with bile exposure severity [3, 7, 12].

Vascular congestion and edema, both indicators of acute or subacute mucosal injury, were also significantly more prevalent in the DGR group. These changes reflect increased mucosal permeability and microvascular damage due to exposure to bile acids and lysolecithin, which are known to disrupt the gastric barrier function [1, 3, 7]. Gastric mucosal damage induces mast cell degranulation and a release of vasoactive mediators, such as histamine, leading to vascular congestion and lamina propria edema [7, 13]. Interestingly, Zhang et al. reported a paradoxical inverse relationship between vascular congestion and reflux severity in their pediatric cohort, suggesting that acute hyperemia may diminish as damage becomes chronic [7]. Nonetheless, in our cohort, congestion remained a consistent finding and was an independent predictor of DGR.

Fibrosis was another striking finding in our study. Although this feature is more commonly associated with chronic injury in adults—particularly in the setting of long-standing bile reflux or post-surgical changes—its presence in a significant portion of our pediatric DGR group suggests that prolonged mucosal exposure to bile can initiate remodeling and early fibrotic transformation, even in children [14]. This aligns with reports of periglandular fibrosis in pediatric DGR patients with longer symptom duration [3, 7, 12].

The relationship between *H. pylori* and bile reflux gastritis remains controversial. While *H. pylori* infection may promote bile reflux by increasing gastrin secretion and reducing antral motility, the alkaline environment created by bile and disruption of the mucosal barrier may, in turn, hinder *H. pylori* colonization or even exert direct bactericidal effects due to high concentrations of bile acids [1]. Studies on *H. pylori* prevalence in patients with bile reflux gastritis have yielded conflicting results, with some reporting reduced infection rates and others finding similar or even higher rates compared to the general population [15–18]. In our study, the infection rate of *H. pylori* in the DGR group was comparable to the control group.

A major distinction between bile reflux gastritis and *H. pylori* gastritis lies in the nature of the inflammatory response. While *H. pylori* infection is typically associated with prominent lymphoid aggregates, neutrophilic activity, and nodularity, bile-induced injury tends to exhibit minimal inflammation and instead features reactive epithelial and stromal/structural changes [12]. This was reflected in our cohort, where inflammatory parameters did not significantly differ between DGR and control groups. Furthermore, the inflamation was more severe, the severity of activity was higher, and lymphoid aggregates were more common in patients with *H. pylori* compared to patients without *H. pylori.*

Atrophic changes and intestinal metaplasia are frequently reported in chronic adult cases, especially post-gastrectomy or with long-standing reflux [5, 6, 19, 20]. Intestinal metaplasia is a type of adaptive change of the gastric mucosa due to the local microenvironmental changes to the intestinal tract. These changes are rarely observed in children, likely due to the shorter duration of exposure and higher mucosal regenerative capacity [21]. Indeed, in our study, neither intestinal metaplasia nor significant glandular atrophy was observed.

Currently, there is no universally accepted gold standard for diagnosing DGR disease in children. Although various diagnostic approaches such as gastroscopy with gastric juice aspiration, hepatobiliary scintigraphy, esophageal impedance-pH testing, and fiberoptic bilirubin monitoring are used, none of these methods are universally standardized, practical or objective enough to serve as definitive diagnostic tools [1]. In our study, we selected patients with gastritis findings who also exhibited the presence of thick bile or bile stains during endoscopy, which is consistent with common practices in the diagnosis of DGR. While we acknowledge the lack of objective quantification, this approach allowed us to create a more homogeneous patient group for histopathological evaluation. Although we cannot claim that the distinct histopathological differences observed in our study provide a definitive differential diagnosis, we believe that histopathological findings identified alongside endoscopic observations in patients with non-specific symptoms could be useful in the differential diagnosis of DGR.

The treatment of duodenogastric reflux disease (DGRD) in pediatric patients lacks a standardized protocol, with commonly used options including ursodeoxycholic acid (UDCA), proton pump inhibitors (PPIs), and sucralfate [3]. Studies in adults have shown mixed results: one found no added benefit of combining UDCA with sucralfate, while another reported greater symptom relief with sucralfate compared to rabeprazole and a control group [22, 23]. In pediatric cases, combination therapies involving cisapride, sucralfate, and omeprazole have shown variable outcomes, with some patients requiring surgical intervention [11]. In our practice, pediatric patients with DGR are treated with a combination of PPIs and UDCA, resulting in a recovery rate of 78.3% (unpublished data). Pediatric patients with histopathological findings consistent with DGR may benefit from combination therapy.

### Strengths and limitations

A major strength of our study is the homogeneity of the patient groups and the blinded re-evaluation of biopsy samples by a single pathologist, which minimized interobserver variability. In addition, we reevaluated the endoscopic images and reports, selected patients with duodenogastric reflux and gastritis findings and also, excluded patients with known *H. pylori* infection and other confounding gastrointestinal diseases, allowing us to more accurately isolate the histological impact of bile reflux.

However, several limitations should be acknowledged. First, this was a retrospective study and relied on endoscopic visualization of bile for DGR diagnosis, without objective quantification (e.g., 24-h bile monitoring). Second, long-term clinical follow-up or assessment of progression (e.g., to atrophy or metaplasia) was not available. Finally, although we applied logistic regression analysis to assess predictive value, the findings should be validated in prospective studies with larger cohorts.

## Conclusion

In conclusion, our study supports and extends the growing body of evidence suggesting that pediatric DGR is associated with distinct histopathological changes resembling those described in adult bile reflux gastritis. Recognition of foveolar hyperplasia, congestion, edema, and fibrosis in gastric biopsies should prompt consideration of DGR, especially in children with non-specific upper gastrointestinal symptoms and bile detected during endoscopy.

## Data Availability

No datasets were generated or analysed during the current study.
